# Supplementary material for: A mathematical model for the dependence of keratin aggregate formation on the quantity of mutant keratin expressed in EGFP-K14 R125P keratinocytes
Source: PLoS One. 2021 Dec 28;16(12):e0261227. doi: 10.1371/journal.pone.0261227 (PMC8714116; doi:10.1371/journal.pone.0261227)
Supplement: S1 Document — (PDF) [file pone.0261227.s001.pdf]

Supplementary Information

October 30, 2021

1 Parameter Value Estimation

On Table 1 of the main text, we present the reaction rates used in the simulation. This table contains the reaction rates that have either been fitted to experimental data (Portet et al 2015) or used in a previous work by the authors (Gouveia et al 2020). In the present work we aim at a simplified description of keratin aggregate formation. Therefore, we use mass-action kinetics, which is suitable to demonstrate qualitatively that aggregates result from asymmetric binding between WT and mutant keratin. As in Portet et al 2015 the authors simulate keratin dynamics using for some reactions the Michaelis-Menten kinetics, in the present work we will use the reaction rates corresponding to their low concentration regimes.

In the model introduced in Gouveia et al 2020, $k_M^{SP/PF}$ were functions of space, depending on the distance to the cell nuclear membrane. The corresponding value of these reaction rates in the present work will therefore be the spatial average of those functions within the cytoplasm. For example,

$$k_M^{SP} = \frac{\int \eta(x,y) k_M^{SP}(x,y)\,\mathrm{d}x\,\mathrm{d}y}{\int \eta(x,y)\,\mathrm{d}x\,\mathrm{d}y}$$

is the average value of the $k_M^{SP}(x,y)$ function in Gouveia et al. 2020 weighted by the level-set function $\eta(x,y)$ that has the value $\eta \approx 1$ in the cytoplasm and $\eta \approx 0$ everywhere else.

2 Alternative Models

In this section we present two additional models for the reaction network of the keratin cycle in cells expressing both WT and mutant keratin 14. These models are simpler than the one introduced in the main text, and we will show why they both fail in describing the results observed experimentally, thus concluding that the asymmetric binding of WT and mutant keratin is likely present in keratin aggregate formation.

2.1 Model I

This first reaction network model analyzed (see Figure 1 left) considers the symmetrical aggregation of WT and mutant keratin. In non-dimensional form, it contains 7 variables: $s_W, s_M, p_W, p_M, f_W, f_M$ describing the same quantities as in the main text. The concentration of aggregates in the cytoplasm is described by the single variable a. The aggregates are formed, by hypothesis, by adding WT and mutant soluble keratin in equal proportions with a rate $k_{agg}S_W S_M$. The conclusions reached with this model are independent of the type of keratins that bind to form aggregates, i.e. if aggregates are formed by the binding of soluble WT and mutant keratins (as in Figure 1) or by WT and mutant particulate keratins. In its non-dimensional form, the system of equations that models the reaction network is:

$$\frac{\mathrm{d}s_W}{\mathrm{d}\tau} = -\lambda_W^{SP} s_W + p_W + f_W + \frac{1}{2}a - \lambda_{agg} s_W s_M$$
$$\frac{\mathrm{d}s_M}{\mathrm{d}\tau} = -\lambda_M^{SP} s_M + p_M + f_M + \frac{1}{2}a - \lambda_{agg} s_W s_M$$
$$\frac{\mathrm{d}p_W}{\mathrm{d}\tau} = \lambda_W^{SP} s_W - p_W - \lambda_W^{PF} p_W$$
$$\frac{\mathrm{d}p_M}{\mathrm{d}\tau} = \lambda_M^{SP} s_M - p_M - \lambda_M^{PF} p_M$$
$$\frac{\mathrm{d}f_W}{\mathrm{d}\tau} = \lambda_W^{PF} p_W - f_W$$
$$\frac{\mathrm{d}f_M}{\mathrm{d}\tau} = \lambda_M^{PF} p_M - f_M$$
$$\frac{\mathrm{d}a}{\mathrm{d}\tau} = \lambda_{agg} s_W s_M - a$$

This model has only one unknown parameter, λ_{agg}. We explored the stationary state of this system of equations for different values of λ_{agg} and fractions of mutant keratin. In Figure 1 right, we plot the fraction of mutant keratin for which the value of Q is maximum as a function of λ_{agg}. For values of λ_{agg} in the range $10^{-2} < \lambda_{agg} < 1$, the maximum value of Q is located at $\chi_M^{max} = 1$, i.e. for cells that contain only mutant keratin. By increasing λ_{agg}, the maximum starts shifting to lower values until it stagnates at $\chi_M^{max} \approx 0.5$ for large λ_{agg}.

Clearly this model is not able to reproduce what was observed experimentally, as the aggregates have by design equal amounts of WT and mutant keratin. Moreover, it is important to notice that, contrary to the experimental observations, in this model χ_M^{max} is always above 50%, regardless of the value of λ_{agg}. To understand the reason for this, consider a cell with 50% WT and 50% mutant keratin. As the formation of mutant filaments is a slower and less efficient process, the fraction of mutant keratin in the soluble and particulate phases is larger than the fraction of WT keratin in those phases. Therefore, the percentage of both soluble and particulate keratins will increase faster by adding mutant keratin to the system than by adding WT keratin. As the formation of aggregates will depend on the amount of soluble keratin in the system,

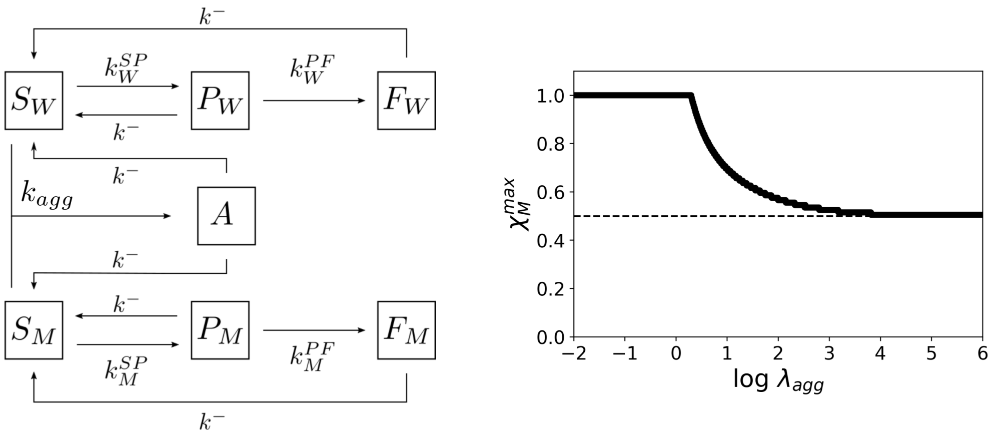

Figure 1: Reaction network diagram for Model I (left). Fraction of mutant keratin at which Q is maximum as a function of $\log \lambda_{agg}$ (right). The dashed line represents χ_M^{max} to which the curve converges as the value of $\log \lambda_{agg}$ increases.

more aggregates will be formed in this model when the amount mutant keratin is above 50% (and a similar conclusion would be reached if the aggregates were formed by adding keratin particles instead).

We have further explored the symmetric 1:1 binding scenario between WT and mutant keratins by considering different soluble to particle reaction rates. Also, in this scenario, we did not identify a region of parameter space which reproduced the experimental observations. Here, we explored the hypothesis that the process of transforming soluble WT keratin into WT particles is slower than its mutant counterpart. With this assumption, we expect an accumulation of WT keratin in the soluble phase that will allow the formation of more aggregates at low mutant keratin fraction. However, we observe experimentally that though cells with 100% mutant keratin are able to form filament networks, they do not have a higher density of keratin fibers than the cells that only expresses WT keratin. Therefore, for the model to truly reproduce the results observed experimentally, we also need to verify that the total amount of fibers ($F_W + F_M$) is either approximately equal or higher in the WT cells (i.e when $\chi_M = 0$) than in the mutant cells (at $\chi_M = 1$).

This model's reaction network the same as before (Figure 1) but instead of only varying λ_{agg} we will also explore how different values of $\beta_{SP} = k_{SP}^W / k_{SP}^m = \lambda_{SP}^W / \lambda_{SP}^m$ affects the formation of keratin aggregates.

In Figure 2A we can observe that there is a region of values for the parameters β_{SP} and λ_{agg} where the maximum amount of aggregates is at $0.10 < \chi_M^{max} < 0.30$. When $\beta_{SP} > 0.1$ the system behaves very similarly to what was observed above: in that regime, as we increase the value of λ_{agg} the maximum of aggregates goes from being located at $\chi_M^{max} \approx 1$ to $\chi_M^{max} = 0.5$. In this region,

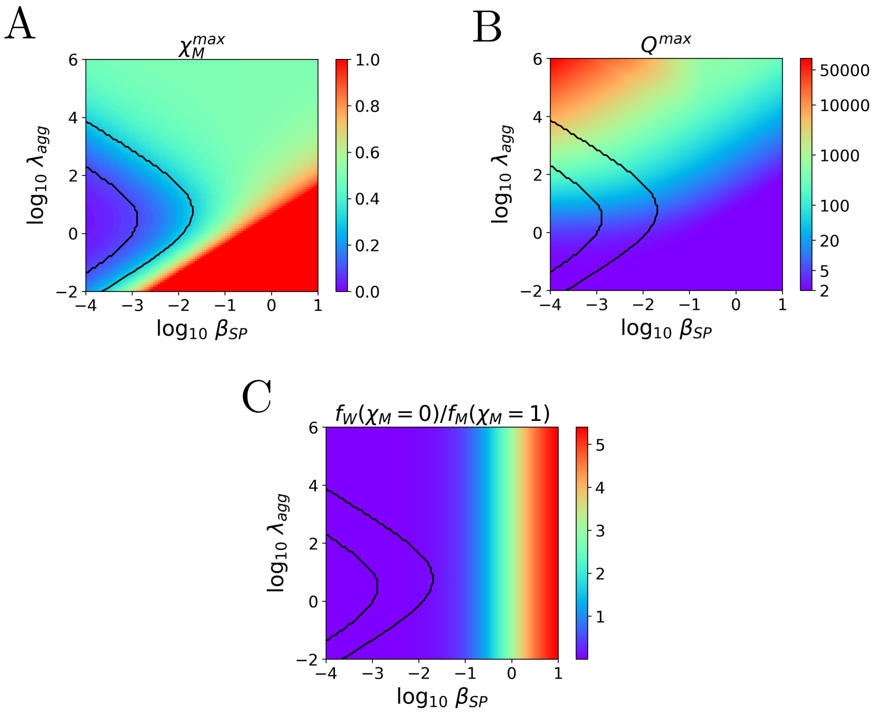

Figure 2: Value of χ_M^{max} (A) and Q^{max} (B) as a function of λ_{agg} and β_{SP}. In (C) we represent the ratio between the fiber fraction of WT cells and fiber fraction in cells with 100% mutant keratin. The black curves bound the region where $0.10 < \chi_M^{max} < 0.30$.

the equilibrium fraction of WT keratin in the soluble phase is still smaller than the equilibrium fraction of mutant keratin in the soluble phase, and therefore the formation of aggregates will occur faster at high concentrations of mutant keratin. As λ_{SP}^W gets smaller (for a fixed value of λ_{SP}^M), we see that for a limited range of λ_{agg} the maximum of aggregates happens with $\chi_M^{max} < 0.5$. In this domain, there is a higher accumulation of WT keratin in the soluble phase which means that the amount of soluble mutant keratin required to promote aggregation will be smaller and, when $\beta_{SP} \approx 10^{-4}$ we have aggregate formation with a fraction of mutant keratin in the system below 50%.

In Figure 2B we see the value of Q^{max} as a function of the same parameters. Inside the region delimited by the black lines (corresponding to $0.10 < \chi_M^{max} < 0.30$) we observe large accumulation of aggregates for $\lambda_{agg} > 10$, according to what is expected from experiments. However, when we look at the ratio of the amount of fibers in cells expressing 100% WT keratin and in cells expressing

100% K14-R125P mutant (Figure 2C), we see that if $\lambda_{SP}^{W} < 0.1\lambda_{SP}^{M}$ (i.e. in the region where we observe $\chi_{M}^{max} < 0.50$) the model predicts that the fiber network of mutant cells is one order of magnitude denser than the fiber network of WT cells. Thus, we conclude that a symmetric binding mechanism between WT and mutant soluble keratin cannot explain the results observed in experiment, even if we consider that the reaction where WT particles are formed is much slower than its mutant counterpart. In this case, we see that by slowing down formation of WT particles process we are creating a bottleneck and limiting the formation of WT keratin fibers.

2.2 Model II

To address the difficulties identified by Model I in replicating the results obtained experimentally, a new reaction network model was hypothesized (shown in Figure 3 left). Analogously to Sun et al 2015, in this model we consider that aggregates are initially nucleated by both mutant and WT keratin through a process with reaction rate k_{nuc}. These aggregates can then grow by the addition of both WT and mutant keratin particles in the soluble phase, with a rate $k_{W/M}^{agg} S_{W/M}(A_W + A_M)$, where k_{W}^{agg} and k_{M}^{agg} are not necessarily identical. The equations that model this reaction network are given by (in non-dimensional form):

$$\frac{\mathrm{d}s_W}{\mathrm{d}\tau} = -\lambda_{W}^{SP} s_W + p_W + f_W + a_W - \lambda_{nuc} s_W s_M - \lambda_{W}^{agg} s_W (a_W + a_M)$$
$$\frac{\mathrm{d}s_M}{\mathrm{d}\tau} = -\lambda_{W}^{SP} s_M + p_M + f_M + a_M - \lambda_{nuc} s_W s_M - \lambda_{M}^{agg} s_M (a_W + a_M)$$
$$\frac{\mathrm{d}p_W}{\mathrm{d}\tau} = \lambda_{W}^{SP} s_W - p_W - \lambda_{W}^{PF} p_W$$
$$\frac{\mathrm{d}p_M}{\mathrm{d}\tau} = \lambda_{M}^{SP} s_M - p_M - \lambda_{M}^{PF} p_M$$
$$\frac{\mathrm{d}f_W}{\mathrm{d}\tau} = \lambda_{W}^{PF} p_W - f_W$$
$$\frac{\mathrm{d}f_M}{\mathrm{d}\tau} = \lambda_{M}^{PF} p_M - f_M$$
$$\frac{\mathrm{d}a_W}{\mathrm{d}\tau} = \lambda_{nuc} s_W s_M + \lambda_{W}^{agg} s_W (a_W + a_M) - a_W$$
$$\frac{\mathrm{d}a_M}{\mathrm{d}\tau} = \lambda_{nuc} s_W s_M + \lambda_{M}^{agg} s_M (a_W + a_M) - a_M$$

This model introduces two additional reaction rates λ_{W}^{agg} and λ_{M}^{agg} for which there is no measured value available in literature. We solved the system of equations and explored their steady state for different values of λ_{W}^{agg} and λ_{M}^{agg}. The value of λ_{nuc} was fixed at 10^3, a typical reaction rate for aggregation according to the results of the model in the main text (though a similar behavior was observed for a wide range of λ_{nuc} values).

In Figure 3 right we represent the value of χ_{M}^{max} as a function of the value of the two aggregation rates. The diagram is essentially divided in 3 main regions

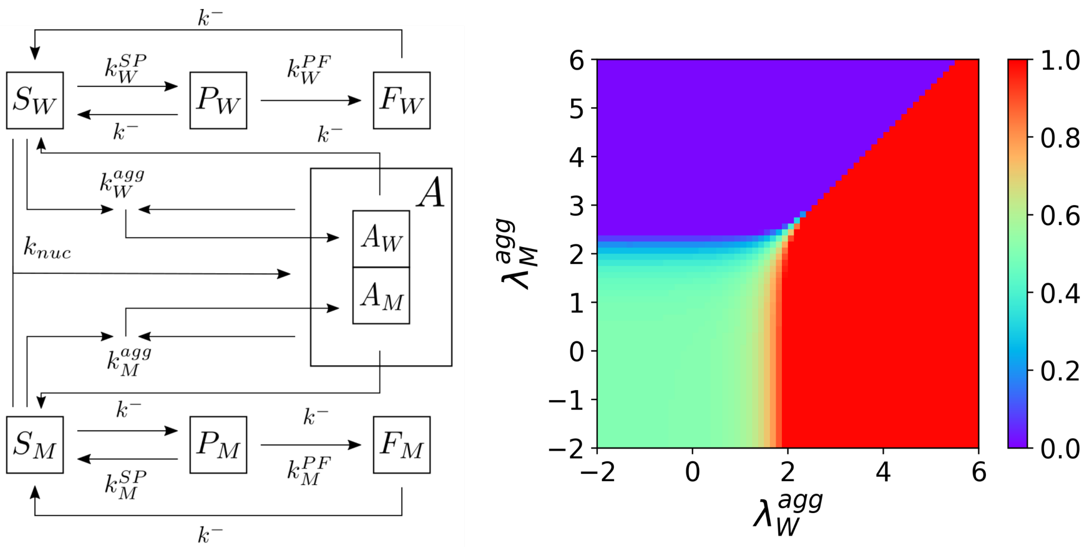

Figure 3: Reaction network for Model II (left). Fraction of mutant keratin at which Q is maximum as a function of λ_W^{agg} and λ_M^{agg} (right).

with very sharp transitions between them. The light green region corresponds to results where $\chi_M^{max} \approx 0.5$, in the red region $\chi_M^{max} \approx 1$ and in the purple region $\chi_M^{max} \approx 0$. The green region is defined by $\log \lambda_W^{agg} < 100$ and $\log \lambda_M^{agg} < 100$. Here, the aggregation constants are small, and the aggregates grow by the nucleation process as in Model I. In the purple region the reaction rate for WT aggregation is larger than the reaction rate for mutant aggregation. Therefore, once there is a small amount of keratin that permits the formation of the aggregation nuclei, the WT keratin is able to attach to those nuclei and grow the aggregate. In this situation, the higher the concentration of WT keratin, the larger the fraction of aggregates in the cell. As a consequence we obtain χ_M^{max} at a concentration of mutant keratin vanishingly small. In the red region the similar reasoning can be made, and the maximum aggregate concentration is reached with a very small quantity of WT keratin. For this model, aggregates can therefore be asymmetric, but very small fractions of mutated keratins can give rise to overwhelming aggregate formation, which is clearly not verified experimentally.

3 Aggregates vs Particles

In the model we monitor the observed accumulation of granular keratin forms (particles and aggregates) by calculating the ratio between these forms and

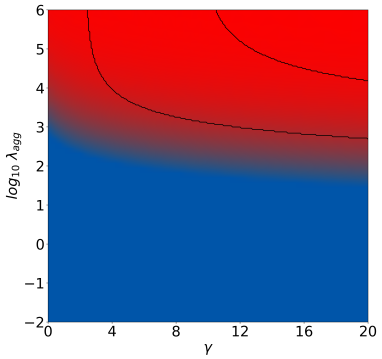

Figure 4: Keratin forms at the WT-mutant keratin composition that maximises Q. Fraction of aggregates is represented in red, keratin particles in blue and keratin filaments in green.

keratin filaments,

$$Q = \frac{a_W + a_M + p_W + p_M}{f_W + f_M} \ ,$$

in the stationary state. In Figure 8 of the main text we observed that a relatively small fraction of mutant keratin leads to the accumulation of these forms. In Figure 4 we explore the fraction these different keratin forms at the composition that maximises Q. Namely, for the mutation fraction χ_M^{max} (which depends on the values of λ_{agg} and γ), we calculate the fraction of keratin in the particle $(p_W + p_M)$, aggregate $(a_W + a_M)$ and filaments $(f_W + f_M)$ forms. A color in RGB is assigned to each corresponding point in Figure 4, such that the red represents the fraction of aggregates, blue the fraction of keratin particles and green the fraction of keratin filaments (normalised by the sum $a_W + a_M + p_W + p_M + f_W + f_M$).

We observe a dramatic accumulation of keratin aggregates in the region of parameter space where Q^{max} reaches the highest values. Therefore, the model suggests that when Q^{max} is in the $\approx 10 - 100$ range, these high values are achieved by the accumulation of keratin aggregates, which result from the binding of WT and mutant keratins.

4 Sensitivity Analysis

We have carried out a full sensitivity analysis of the model regarding to variations of the parameters λ_W^{SP}, λ_M^{SP}, λ_W^{PF}, λ_M^{PF}, λ_{agg}, γ and χ_M. With this aim we carried out positive and negative variations of 10% as well as doubling and halving each parameter, and calculated the corresponding variation in Q. We started from the state with $\lambda_{agg} = 1.0 \times 10^4$, $\gamma = 5.0$ and $\chi_M = \chi_M^{max} = 0.13$. For these parameters we have $Q^{max} = 37$ The percentual variations of Q after nudging each parameter can be seen in the following table.

Percentual Variation in Q

Variable	Variation +10%	Variation −10%	÷2	×2
λ_W^{SP}	+1.29%	−3.19%	−34.13%	−35.20%
λ_M^{SP}	−0.65%	−1.75%	−73.50%	−18.94%
λ_W^{PF}	−4.73%	+5.26%	+33.86%	−32.61%
λ_M^{PF}	−4.80%	+5.54%	+40.69%	−30.18%
λ_{agg}	+4.76%	−5.23%	−32.59%	+33.87%
γ	+1.92%	−8.80%	−92.17%	−14.88%
χ_M	−4.31%	−7.56%	−94.15%	−59.05%

We observe that Q has a relatively large sensitivity regarding all parameters in the model, with a percentual variation on the same order of magnitude as the percentual variation of most parameters. In fact, for the larger variations of the reaction rates tried (by doubling or halving the value each parameter), we observe typically a large variation in Q (by approximately 30%). The sensibility of Q is even larger to variations in the parameters γ and χ_M.

5 Total keratin content in the EGFP K14 cell lines

All the EGFP K14 cell lines (R125P or mutant) were tested for a potential change in total keratin content as consequence of the EGFP K14 construct expression. As shown in Figure 5, after imunoblotting with a pan-keratin antibody (AE1/AE3, Agilent Dako, USA), we found no major difference in keratin expression pattern between the different cell lines.

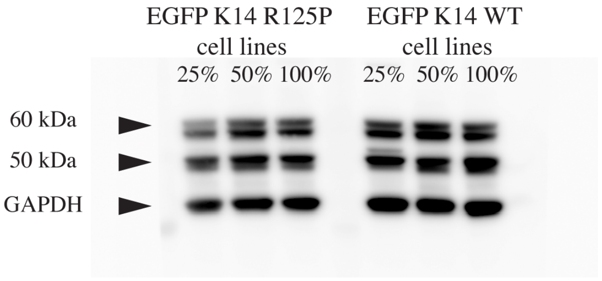

Figure 5: Imunoblotting of total cell extracts against a pan-keratin antibody revealed no major differences amongst the EGFP K14 cell lines (the EGFP-K14 R125P sample has been loaded a little bit less than all the other samples; see GAPDH for comparison, the loading control). A faint additional band is visible only in the 25% EGFP-K14 WT cell line, which is not present in any of the other samples.
