# Supplementary figures and images for: A mathematical model for the dependence of keratin aggregate formation on the quantity of mutant keratin expressed in EGFP-K14 R125P keratinocytes
Source: PLoS One. 2021 Dec 28;16(12):e0261227. doi: 10.1371/journal.pone.0261227 (PMC8714116; doi:10.1371/journal.pone.0261227)

Figure 4

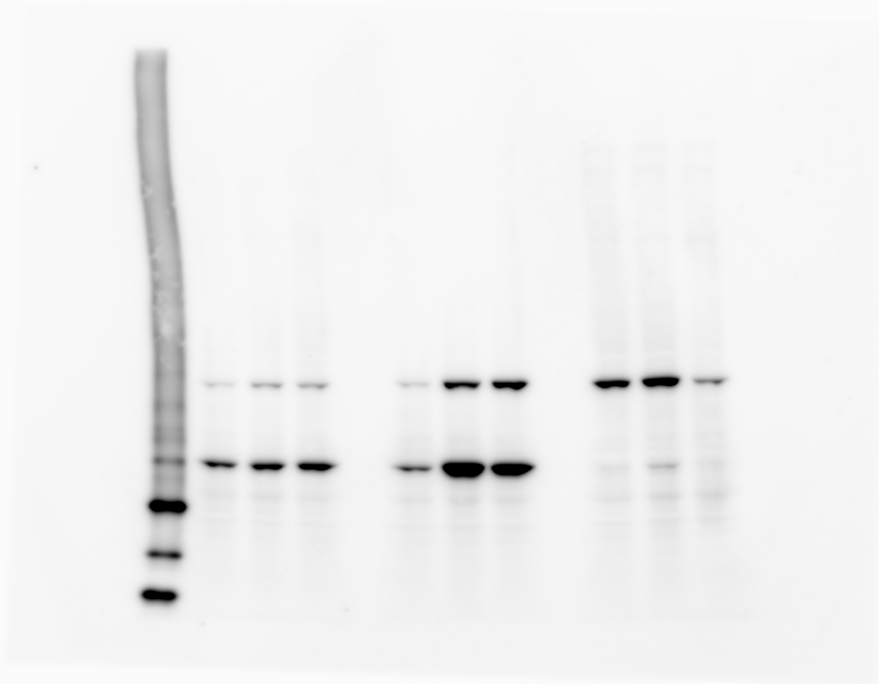

Figure 7

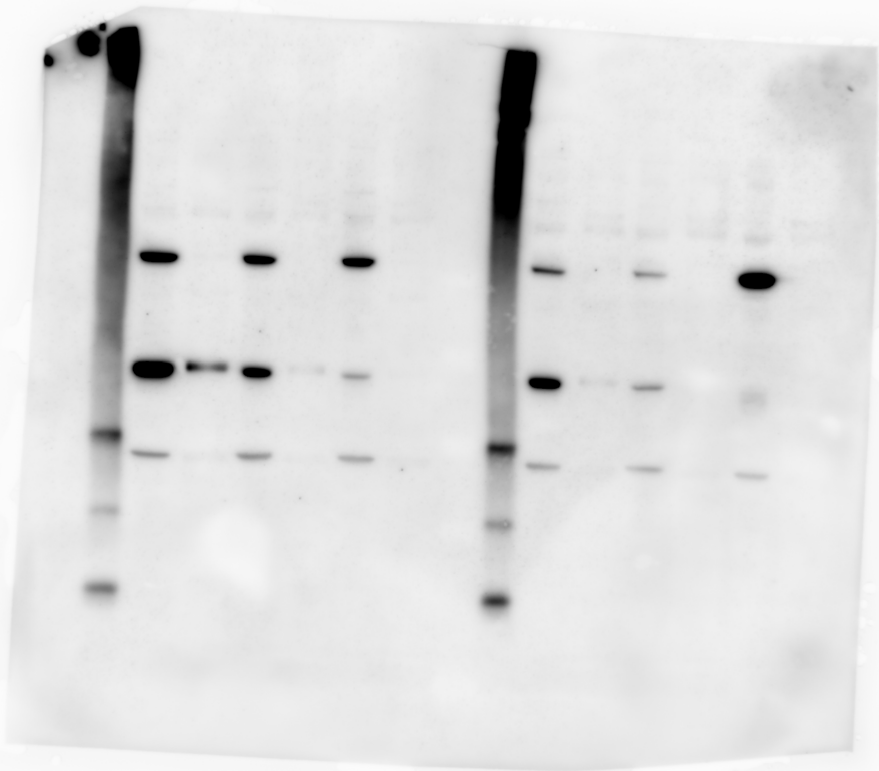

Supplement: S1 Raw images — (PDF) [file pone.0261227.s003.pdf]
