# Supplementary material for: A mathematical model for the dependence of keratin aggregate formation on the quantity of mutant keratin expressed in EGFP-K14 R125P keratinocytes
Source: PLoS One. 2021 Dec 28;16(12):e0261227. doi: 10.1371/journal.pone.0261227 (PMC8714116; doi:10.1371/journal.pone.0261227)
Supplement: Attachment — Submitted filename: Reply.pdf [file pone.0261227.s004.pdf]

We thank the Reviewers for their careful revision and inputs, which we have included in this new extensively altered and improved version of our manuscript. To answer the Reviewers requests we have repeated the experiments, and present a clearer Figure 7 and a fifth figure in the supporting material to quantify the total keratin in these cells. We now provide point-by-point answers to the reviewers' specific comments.

Reviewer #1:

The paper entitled ``A Mathematical Model of the Dependence between Keratin Aggregate Formation and the Quantity of Mutant Keratin Expressed in EGFP-K14 R125P Keratinocytes" presents a combination of experimental and computational results on the formation of keratin aggregates in keratinocytes.

The paper presents interesting results using a broad collection of methods from cell biology, biomechanics and biomathematics; however, some results are over-interpreted or all data are not presented or properly explained making some rationale hard to follow. The paper needs revisions before publication.

-Total amount of keratin (endogenous + EGFP K14): Have you verified the total amount of keratin in the different conditions (25%, 50% and 100%)? Does any condition induce an increase/decrease of the total amount of keratin in cells?

The total amount of keratin 14 in the different conditions (25%, 50% and 100%) is shown on the Western blot in Figure 4. We have now also quantified experimentally the total keratin in these cells and observed no major difference in total keratin amongst the EGFP-K14 R125P or EGFP-K14 WT cell lines (see new section in Supporting Information). Moreover, regarding the mathematical model, all parameters are independent of the total amount of keratin except for λ_{agg}, which increases linearly with K_{total}. In Fig 8 we considered that λ_{agg} (and therefore K_{total}) is not a function of χ_M. If K_{total} depended on χ_M, λ_{agg} would vary accordingly. To explore if the conclusions of our calculation would be altered in the latter situation, we repeated the simulations displayed in Figs 8B and 8C considering that K_{total} (and λ_{agg}) decreases by 50% as χ_M increases from 0 to 1. The results are displayed in the following plots:

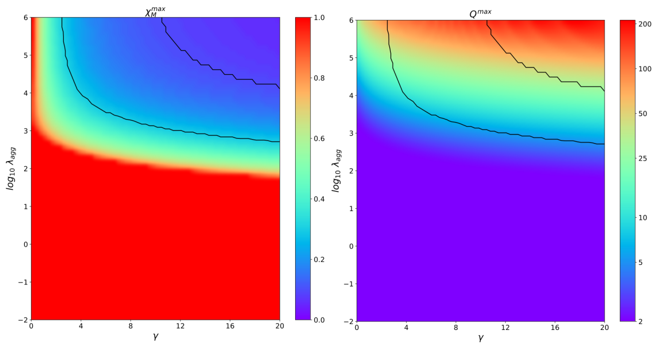

These images do not differ significantly from Figs 8B and 8C and therefore we conclude that including a moderate variation of keratin amount with the fraction of mutant keratin added would not impact our conclusions.

- Line 136 (Definition of insoluble fraction (P)): Specify what does P refer to? filaments + particles + aggregates? endogenous only, EGFP K14 only, mix of endogenous and EGFP K14 material?

The insoluble fraction refers to all insoluble keratin in cells, which is left in the pellet after protein extraction with RIPA buffer (has mild denaturing conditions due to SDS). On Western blot we specifically looked at K14 by immunoblotting, as this is the protein we are interested in. Under the extraction conditions we used (RIPA buffer followed by high salt extraction), the RIPA supernatant (marked S on the gel in Figure 7) should contain most of the cell keratin, while the RIPA insoluble fraction (the pellet, marked as P in Figure 7), which was subsequently further treated/extracted with a high salt buffer, should contain only small, leftover amounts of keratin. As shown in Figure 7, the latter is true for almost all the EGFP cell lines, be it WT or mutant, apart the 25% mutant EGFP-K14 R125P cell line. The RIPA pellet of the 25% mutant contains considerable amounts of endogenous K14 WT, as well as a smaller amount of the EGFP-labelled K14 R125 protein. At the same time no or very little K14 (endogenous or EGFP-labelled) is present in the pellets of the EGFP-K14 WT as well as the 50% and 100% K14 R125P cell lines. We hypothesized that this may be due to the larger number of cells with aggregates in the 25% EGFP-K14 R125P mutant in comparison to the 50% and 100% one. Following this line of thought we reasoned that the RIPA buffer extraction does not manage to solubilize the numerous aggregates extracted from the 25% EGFP-K14R125P mutant, while it solubilizes the few aggregates seen in the 50% and 100% mutants. We have revised this section of the manuscript with a new Figure 7 as requested by reviewers. We have also revised the Materials and methods section regarding the extraction process, as it was not sufficiently well described.

-Interpretation of γ : γ is used in the model to describe the asymmetric formation of aggregates from wild and mutant particles. From the equations of the model we have: if $\gamma<1$ (resp. $\gamma>1$), the per capita rate of formation of A_W from P_W is smaller (resp. larger) than the per capita rate of formation of A_M from P_M for the same concentrations of the other type of particles P_M or P_W. The authors write ``γ is used to represent the difference in size between typical 'WT and mutant particles that are able to bind and form aggregates.". This sentence is misleading. A simple sentence in the mathematical model section as " γ is used to represent the asymmetric formation of aggregates from wild and mutant particles" should be enough. Related to this issue, problem with the interpretation of results on page 11 and line 427.

Reviewer #2 also raised this point. We agree and have replaced the sentence by the following: "We use the parameter γ to reflect the asymmetric formation of aggregates from WT and mutant particles, which is not necessarily in a 1:1 ratio. A possible asymmetric mechanism of aggregate formation is by binding WT and mutant particles of different typical sizes."
We also corrected the discussion in page 10 and in the conclusions of the document accordingly. The discussion in page 11 does not mention the particle sizes, so it still holds.

- Define K_{Total} in line 206.
Done

- Define the total fraction of mutant keratin in cells χ_M in line 212:
$\chi_M=s_M+p_M+A_M+f_M$ with $\chi_W+\chi_M=1$
Done

- Results of models are given at stationary state. Specify the criterion used to detect the fact the solutions of the model have reached the steady state value. That is missing in section ``Numerical methods" starting on line 213.

We have now included the stationary state criterion in the Numerical Methods section: "The simulations were run until the stationary state. We considered that the stationary state is numerically reached when the absolute value of the time derivatives of all keratin fractions were below 1×10^{-10}~s^{-1}."

- Remove the sentence ``Due to the size of the system of equations and to its non-linearity, it is not possible to find the stationary state solution analytically". By reducing the dimensional system to a 6-dimensional system (using the conservation of law on the total amount), some expression might be obtainable but an only numerical approach as followed in the paper is enough.
Done.

- Figure 4: Does the total amount of keratin in cells change when mutants are added?

We performed an additional Western blot for the reviewers and imunoblotted it with a pan-keratin antibody (see new section in Supporting Information and Figure 5 therein). As shown, no major difference in total keratin amongst the EGFP-K14 R125P or EGFP-K14 WT cell lines was be observed.

- Figure 5: At least 35 cells are used to obtain the measurements of cell stiffness. Nothing is mentioned on how these cells are sampled (selected) for each condition. Does the samples used for stiffness measurements are representative of results shown in Figure 6?

Under Materials and Methods, Optical Tweezers and indentation experiments, we described how these experiments are performed. The cells (samples) are not selected at all but measured at random. In order to allow access to cells with the laser/bead, the cells need to be seeded quite diluted and measurements are then performed on single cells (not groups of cells). We explain in the manuscript: “A bead was trapped by optical tweezers and positioned near a single, non-confluent cell, facing the cell side where the cell membrane was most vertical, i.e., the side without extensive lamellipodia.” Therefore, Figure 6 is not representative for the state of confluence used for the optical tweezers measurements, but it is representative for the type (appearance) of cells that were measured.

- Figure 5: What about the stiffness of cells expressing only endogenous keratin? Does the addition of EGFP K14 construct change the cell stiffness?

The stiffness of the parental cell line (NEB1 cells) expressing only endogenous keratin was measured in the paper we recently co-published (Zemljic-Jokhadar et al., PLOS One 2020). However the experimental set up of the optical tweezers in that set of experiments is different, so this kind of comparison is difficult to make based on the different nature of the two sets of data. We have not measured the stiffness of the NEB1 cells again under the

new experimental set up.

- Figure 5: Specify the values referred by stars.

We have replaced the stars with values in the revised Figure 5.

- Figure 5: Change the title of this figure. This title is not representative of the information displayed in the figure.

We have now changed the title with “Correlation between cell stiffness and the expression rate of the EGFP-K14 constructs”

- Figure 6: Add the results for EGFP K14 WT as a control case.

In the EGFP K14 WT cell lines there are no cells with aggregates (please see also Gouveia et al. IJMS 2020). Regardless of the amount of EGFP-K14 WT construct expressed, this does not cause the phenomenon of keratin aggregation. In general, keratin aggregation is linked specifically to keratin mutants (as described in manuscript), and in particular when case of severe keratin mutations, like the K14 R125P mutation.

- Line 283, "..however the quantity of aggregates did not correlate with the expression level of EGFP-K14 R125P protein." How do the authors get this conclusion? No information on the quantity of aggregates is provided, only information about the number of cells with aggregates is available. Rewrite this sentence.

We thank the reviewer for this comment. We have revised the sentence as: “Lower cell stiffness did correlate with the presence of keratin aggregates, however the number of cells with keratin aggregates was inversely proportional to the expression level of EGFP-K14 R125P protein.”.

- Figure 7: Add the results for EGFP K14 WT as a control. Usually IF proteins are found mostly assembled in the insoluble fraction. However, for all conditions in presence of mutant, the soluble fraction is larger than the insoluble one. (As previously mentioned, more details should be given on what is the insoluble fraction.)

These results were added to Figure 7. We have now explained our total protein cell extraction method in detail; please see Materials and methods. As we used RIPA buffer extraction, it is normal that majority of cell keratin ends

up in the RIPA buffer supernatant (marked S in Figure 7), while very little to none in the pellet (marked as P in Figure 7).

- In line 301 "..due to it being sequestered within keratin aggregates, which may be of lower solubility than the filaments.". The authors interpret the results from Fig 7 by speculating that aggregates might have a lower solubility than filaments. However, the disassembly rate k_{-} is assumed to be the same for all (particles, aggregates and filaments) in the model.

The k_{-} rate describes the de-polymerization/de-aggregation rate of the keratin filaments/agglomerates in the cell, one monomer at a time. As explained above, in the solubilization process that takes place during keratin extraction compounds are added to force keratin solubilization, and therefore we expect the solubilization mechanism to be necessarily different from the one described by the k_{-} rate.

- Line 319: The definition of Q as the observable fraction of keratin aggregate in the cell cytoplasm is a little misleading. The equation defining Q is the ratio of the proportion of particulate material to that of filamentous material in cells.

This point is now clarified in the new version.

- Line 320: Typo in the definition of Q.
Corrected.

- Add a reference to a figure to the sentence starting on line 351.
Added.

- On Line 361, give a proper reference to a figure or other instead of just "see above".
Done.

- Validation of the model by experiments (paragraph starting on line 349): Figure 6 deals with the number of cells with aggregates, Figure 7 deals with the soluble and insoluble fractions (the insoluble fraction is not clearly defined yet) of endogenous and EGFP K14. But no clear experimental data are shown about the quantity of aggregates in a cell. Add information or explanation or rewrite.

There is a clear variability in the experimental observations that the model was not designed to reproduce. Experimentally, at present we do not have a way to quantify the amount of keratin aggregates in an individual cell, and we can only detect how many cells at a given time display the phenomena of keratin aggregation.

In the mathematical model we have constant values for the rates of keratin polymerization and de-polymerization. With the model we conclude that the proposed asymmetric aggregation between WT and mutant provides a peak in aggregation at low mutant fractions as is verified in the experimental assays. Experimentally, though, we observe a large variability of aggregation levels from cell to cell, as it is clear from the data in Figure 6.

Interestingly, in the mathematical model we show that a relatively small variation in the parameters (see the new sensitivity analysis in the supporting information) can give rise to a significant variation in *Q.* We also observe in Figure 8A that the aggregation peak is rather narrow. We hence expect to obtain a wide range of aggregation levels if we simulated a group of cells with equal χ_M but where the reaction rates may vary from cell to cell by a few tens of percent. This study of variability of reaction rates is outside the scope of the present work, which is to propose the mechanism of asymmetric aggregation. To clarify, we added a sentence at the end of subsection Endogenous K14 WT is sequestered in the cell pellet.

"This model will not look into the variability in aggregate concentration within a population of cells with similar keratin mutant levels (which will be the focus of a future work), but it will explore possible aggregation mechanisms that allow for the existence of a peak in aggregation at low mutant keratin fractions, as observed in Figures 6 and 7."

- Further validation of the model by experiments: From Figure 7, information on the total (endogenous + EGFP K14) soluble and insoluble fractions is available. This information is also tractable in the mathematical model. The model should also be able to predict these features too. Knowing the ratio between particulate and filamentous material (defined by Q) is interesting but this information could be accompanied by the distribution of keratin between the soluble and insoluble fractions.

As it is now clearly mentioned in the manuscript, we justify the presence of the insoluble fraction by the accumulation of keratin aggregates. The model typically produces narrow peaks when Q is plotted against χ_M (see Fig 8A). Due to the strong non-linearity of these curves, to achieve quantitative agreement on the amount of keratin aggregates observed, we should incorporate the variability in the reaction rates as discussed in the previous reply. However, as an example, for the parameters for which the sensitivity

analysis was carried out (corresponding to a peak with Q^{max}=37 and $\chi_M^{max} = 0.13$), we obtain the following profile of insoluble (aggregates) vs soluble (all the other phases) keratin:

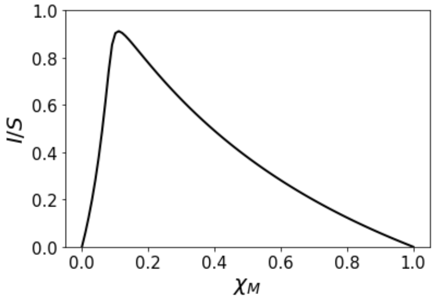

The peak reaches I/S=0.9, indicating approximately equal amounts of aggregates and the remaining keratin phases (at the peak, for these parameters, the keratin in aggregates has a similar concentration as the soluble keratin monomers, with very small concentration of keratin particles and filaments). These proportions are similar to the ones observed in Figure 7 for low mutant keratin concentrations. However, as it is evident from Figure 6, the variability from cell to cell in keratin aggregation will play an important role in the quantitative agreement of experimental and theoretical results. As indicated above, the study of variability of reaction rates is outside the scope of the present work, which is to propose the mechanism of asymmetric aggregation.

To clarify we added a sentence at the end of the conclusions:

"In a future study we will address quantitatively the variability observed in keratin aggregation at the cell level. Introducing variability in the reaction rates of the model will also permit to better explore quantitatively these measured amounts of keratin."

- The experimental results and their interpretation highlight the importance of the solubilization process translated in the model with the term depending on k_{-}. A full sensitivity analysis should be carried out to determine the influence of other parameters (not only λ_{agg} and γ).

As mentioned above, k_{-} is not related to the solubilization process that is carried out in Fig.7. We nevertheless proceeded with the full sensitivity analysis. We added a new section (section 4) in the supporting material with this information. *Q* is very sensitive to variations in all parameters, but in particular to variations in χ_M and γ.

- Line 376: ".. as the more keratin aggregates are present in cells, the lower the stiffness of the cells' cortical area." Similar remark as before, this statement seems to

be an over-interpretation of results.

This sentence has been revised accordingly, just as described in one of the previous comments: “as the larger the number of cells with aggregates, the lower the stiffness of the cells' cortical area.”
Note that the cell stiffnesses plotted on graph are average values of the measurements performed.

Minor remarks:
- Add a reference to the sentence starting on line 36.
Done.

- Typo or missing information on line 82: "(Clontech, , USA)"
Clontech, USA. The comma is extra. Corrected

- Typo on line 420
Done.

- The supplementary material should be polished.
Done. Please see alterations marked in the track-changes document.

Reviewer #2:
The manuscript by Gouveia et. al. entitled “A Mathematical Model of the Dependence between Keratin Aggregate Formation and the Quantity of Mutant Keratin Expressed in EGFP-K14 R125P Keratinocytes” is interesting and well written. The authors investigate how cells with various proportions of mutant keratin and wild type keratin are different. They examine the number of cells exhibiting keratin aggregates, cell stiffness, and the amount of soluble vs insoluble keratin in the cells. Additionally, the authors developed a mathematical model to help explain the surprising experimental results. The paper should be published. I have several comments.

Pg 5 “gamma is used to represent the difference in size …” I think gamma gives the ratio of wt to mutant in the aggregation and stating that would be clearer.

Corrected. Please see reply to Reviewer #1 who has raised this very point.

Fig 5 What do the *** mean? It is not explained in the caption

The stars have now been replaced with the values, and are related to the level of significance.

Fig 6 The arrows are not mentioned in the caption

The arrows are now mentioned in the revised caption.

Pg 9 Five times more WT than mutant but only 18 percent of cells show aggregates. You should be able to approximate how much more WT is in the aggregates. Also is the mutant consistent with the 18 percent – the soluble is much darker than the pellet is it greater than 18 percent (since the pellet is more than just the aggregate)?

Please bear in mind that keratin aggregates are not stable and static like in some other protein aggregation diseases (e.g. Alzheimer), but are dynamic and are constantly being produced and degraded by the cells (see video in supplemental material, where keratin aggregates slowly disappear and filaments are being formed instead). Therefore, such approximations are hard to do.
The RIPA supernatant should contain almost all keratin, however the RIPA pellet can contain smaller amounts of leftover keratin. We assumed that in case of the 25% EGFP-K14 R125P mutant this is mostly due to keratin aggregates, as these were the only obvious potential cause of this.
Notice that, in the new experimental data of Figure 7, we obtained a ratio between WT and mutant keratin in the pellet that is larger than previous 5 to 1. Importantly, all conclusions regarding the asymmetric aggregate formation still hold.

Fig 7 Why change the order in this figure to 25% 100 % 50%? Also the 100 % had the least aggregate but seems to have more pellet than the 50 %?

We did not change the order in Figure 7, this is how samples were loaded on gel and recorded with the camera. We have now provided a new Figure 7, with a less confusing order of samples as well as with the EGFP-K14WT samples. The RIPA insoluble fraction, the pellet, may contain trace amounts of keratin, regardless whether the cells are mutant or wild type. Furthermore, we hypothesized that as there is no or very little keratin present in almost all the EGFP cell lines (be it WT or mutant) apart the 25% mutant, this difference may be due to the presence of a larger number of cells with aggregates, as we do not see much keratin left in the pellet fraction of the 50% and 100%

EGFP-K14 R125P mutants, where also less cells with aggregates were seen. This new figure also contains GAPDH as loading control, so samples were loaded in similar amounts and we do not see that more keratin is present in the pellet of the 100% mutant than the 50% one.

Pg 10 Why is Q not (aW+aM)/(fW+fM)? Why include the particulate keratin? It seems figure 8 would make more sense (or at least in the explanation) if the aggregate keratin was reported from the model as well as Q (or instead).

We chose Q as the ratio between all particulate keratin forms and filamentous forms to be closer to the experimental microscopy visualization of these aggregates. Experimentally it is often observed that these particles integrate existing filaments, making them grow (see Gouveia et al. (2020) International Journal of Molecular Sciences. 21). Hence, both on-pathway keratin particles (p_M and p_W in the mathematical model), and off-pathway larger aggregates (a_M and a_W in the mathematical model) can be visualized experimentally. In practice, however, the peak in Q is determined by the accumulation of aggregates (which can reach concentrations much larger than the on-pathway keratin particules), and the two definitions of Q yield very similar plots for Figure 8 (see reply to next comment).

Pg 10 line 372 "magnitude more keratin in aggregates than in filaments" The model says Q should be that not the aggregates (Q is more than just the aggregates).

We have added a new section in the supporting information that goes into more detail into the fraction of aggregate and particulate keratin at the vicinity of χ_M^{max}. The section also includes a new Figure.

"3. Aggregates vs Particles

In the model we monitor the observed accumulation of granular keratin forms (particles and aggregates) by calculating the ratio between these forms and keratin filaments, $Q = \frac{a_W+a_M+p_W+p_M}{f_W+f_M}$, in the stationary state. In Figure 8 of the main text we observed that a relatively small fraction of mutant keratin leads to the accumulation of these forms. In Figure 4 we explore the fraction these different keratin forms at the composition that maximises Q. Namely, for the mutation fraction χ_M^{max} (which depends on the values of λ_{agg} and γ), we calculate the fraction of keratin in the particle ($p_W + p_M$), aggregate ($a_W + a_M$) and filaments ($f_W + f$) forms. A color in RGB is assigned to each corresponding point in Figure 4, such that the red represents the fraction of aggregates, blue the fraction of keratin particles and green the fraction of keratin filaments (normalised by the sum $a_W + a_M + p_W + p_M + f_W + f_M$).

We observe a dramatic accumulation of keratin aggregates in the region of parameter space where Q^{max} reaches the highest values. Therefore, the

model suggests that when Q^{max} is in the ≈ 10 - 100 range, these high values are achieved by the accumulation of keratin aggregates, which result from the binding of WT and mutant keratins.

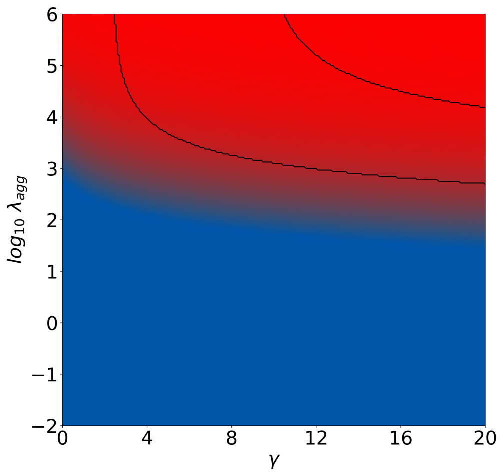

Figure 4: Keratin forms at the WT-mutant keratin composition that maximises *Q*. Fraction of aggregates is represented in red, keratin particles in blue and keratin filaments in green."

More explanation of the stiffness results should be included.

We have now added a few extra lines to make sure that the readers keep in mind that that keratin aggregates are not stable and static like in some other protein aggregation diseases (e.g. Alzheimer), but are dynamic and are constantly being produced and degraded by the cells (see video in supplemental material, where keratin aggregates slowly disappear and filaments are being formed instead). We also pointed out that we have not selected cells with aggregates to then measure cell stiffness. In fact, we did no cell selection whatsoever in that respect - our optical tweezer set up does not include UV light/adequate filters, so all measurements on cells are done at random. The resulting cell stiffness plotted on graphs are average values of the measurements performed

Minor issues:

Pg 6 line 203 concentrations is stationary change to concentrations are stationary

Done.

Pg 10 Q=(….)/(fM+fM) should be (…)/(fM+fW)

Done.

Pg 12 line 420 “model?s”
Done.
